# High Incidence of Diabetes in People with Extremely High High-Density Lipoprotein Cholesterol: Results of the Kanagawa Investigation of Total Checkup Data from the National Database-1 (KITCHEN-1)

**DOI:** 10.3390/jcm8030381

**Published:** 2019-03-19

**Authors:** Kei Nakajima, Ryoko Higuchi, Taizo Iwane, Michi Shibata, Kento Takada, Michiko Sugiyama, Masafumi Matsuda, Teiji Nakamura

**Affiliations:** 1 School of Nutrition and Dietetics, Faculty of Health and Social Services, Kanagawa University of Human Services, 1-10-1 Heisei-cho, Yokosuka, Kanagawa 238-8522, Japan; higuchi-nk3@kuhs.ac.jp (R.H.); iwane-d2k@kuhs.ac.jp (T.I.); sibat.nu@marianna-u.ac.jp (M.S.); takada-dn2@kuhs.ac.jp (K.T.); sugiyama-m@kuhs.ac.jp (M.S.); nakamura-t@kuhs.ac.jp (T.N.); 2 Department of Endocrinology and Diabetes, Saitama Medical Center, Saitama Medical University, 1981 Kamoda, Kawagoe, Saitama 350-8550, Japan; matsudam@saitama-med.ac.jp; 3 Department of Nutrition, St. Marianna University School of Medicine, 2-16-1 Sugao, Miyamae-ku, Kawasaki, Kanagawa 216-8511, Japan

**Keywords:** HDL, diabetes, HbA1c, fasting plasma glucose, body mass index, alcohol consumption

## Abstract

Background: It is unknown whether extremely high high-density lipoprotein cholesterol (HDL-C) has a protective effect against diabetes, which plays a key role in cardiovascular disease. Methods: In a community-based cohort study of 387,642 subjects (40–68 years old) without diabetes, the incidence of diabetes 6 years later was determined according to baseline HDL-C (≤39, 40–49, 50–59, 60–69, 70–79, 80–89, 90–99, 100–109, or ≥110 mg/dL). Results: At baseline, HDL-C ≥100 mg/dL was present in 12,908 subjects (3.3%), who had a better lipid profile and a high prevalence of heavy alcohol consumption and habitual exercise. The incidences of diabetes according to baseline HDL-C were 14.7, 11.2, 7.7, 5.3, 3.8, 2.8, 2.7, 2.5, and 3.5 per 1000 person-years, respectively. The adjusted relative risks (ARRs) for diabetes showed concave relationships with HDL-C, with minima at 80–89 mg/dL. The ARR (95% CI) of the lowest HDL-C category was 1.56 (1.40–1.74) and of the highest HDL-C category was 1.46 (1.18–1.81) (both *p* < 0.001), regardless of alcohol consumption. The latter ARR was higher in men (*n* = 219,047) (2.45 (1.70–3.53), *p* < 0.0001) after adjustment for baseline glycemic index. Conclusion: Both extremely high and low HDL-C represent risks for diabetes, which deserves further study.

## 1. Introduction

Evidence from very large-scale clinical studies indicates that people with a low serum concentration of high-density lipoprotein cholesterol (HDL-C) are at greater risk of cardiovascular disease (CVD) [1,2,3]. Therefore, a high concentration of serum HDL-C has long been considered to be associated with a lower risk of atherosclerosis, consistent with the concept of “the higher the better”. In addition, a lower limit for serum HDL-C, such as 40 mg/dL, is used as a guideline for the prevention of CVD in American and Japanese guidelines [4,5]; however, an upper limit and an optimal concentration of HDL-C have not been determined. Furthermore, although high HDL-C concentrations can be attributed to genomic factors, including *APOA1*, *LCAT*, *ABCA1*, and *CETP* genotypes [6,7,8], and particular lifestyles, such as heavy alcohol drinking [9,10,11] and habitual exercise [12,13,14], the risk associated with high HDL-C and its association with other CVD risks have been poorly characterized.

During the last decade, doubt has been cast on the protective effect of very high HDL-C against CVD [15,16,17]. Large cohort studies have shown that higher mortality rates associated with CVD, including due to myocardial infarction and stroke, are observed in individuals with HDL-C values over 80–100 mg/dL [18,19,20,21,22]. To date, however, conclusive evidence has not been obtained, possibly because of the small proportion of people with extremely high HDL-C in the general population [17], which hampers proper statistical analysis in even quite large samples. Thus, the precise proportion of individuals with very high HDL-C and its underlying etiology have not been determined.

The prevalence of type 2 diabetes, an important risk factor for CVD, has been increasing worldwide. Type 2 diabetes is associated with atherosclerosis and a greater risk of CVD-related mortality, and has a complex, multifactorial etiology involving genetic predisposition, obesity, other endocrine diseases, and an unhealthy lifestyle, including smoking, heavy alcohol drinking, and infrequent exercise [23,24]. In this context, we hypothesized that the incidence of diabetes may be relatively higher in people with extremely high HDL-C. Therefore, we investigated the relationship between serum HDL-C concentration and the incidence of diabetes in a 6-year community-based observational cohort study of 387,642 Japanese people.

## 2. Methods

### 2.1. Study Design

We conducted a composite multidisciplinary study involving the secondary use of mandatory health check data, in Japan, which was termed the “Kanagawa Investigation of the Total Checkup Data from the National Database” (KITCHEN), aimed at elucidating the factors primarily associated with cardiometabolic diseases. The overall study concept and design has been described in detail elsewhere [25]. From 2008, everyone aged 40–74 years living in Japan has been encouraged to undergo an annual specific health check, managed by the Ministry of Health, Labor, and Welfare (MHLW) [25,26]. The current study was of everyone who underwent this specific health check and who was living in Kanagawa prefecture, the population of which (approximately 9.1 million) is the second biggest in Japan. During the period between 2008 (baseline) and 2014, the number of subjects undergoing the standard health check has increased annually in Kanagawa (from 37% to 50% across the entire population of 40–74-year-olds), in parallel with the nationwide trend [25]. Nationally, almost 50% of the population attended a health check in 2014, probably because of political encouragement for people to participate in these checks.

The study protocol was approved by the ethics committee of Kanagawa University of Human Services (No. 10-43) and the MHLW in Japan (No. 121). We received digitally recorded anonymous data from the MHLW, as part of its nationwide program, involving the provision of medical data to third parties [25]. To further protect against the identification of specific individuals, their ages were categorized as 40–44, 45–49, 50–54, 55–59, 60–64, 65–69, or 70–74 years before the datasets were obtained, so that an individual’s precise age at the time of data collection was not identifiable by the researchers.

### 2.2. Subjects

**Figure 1:**
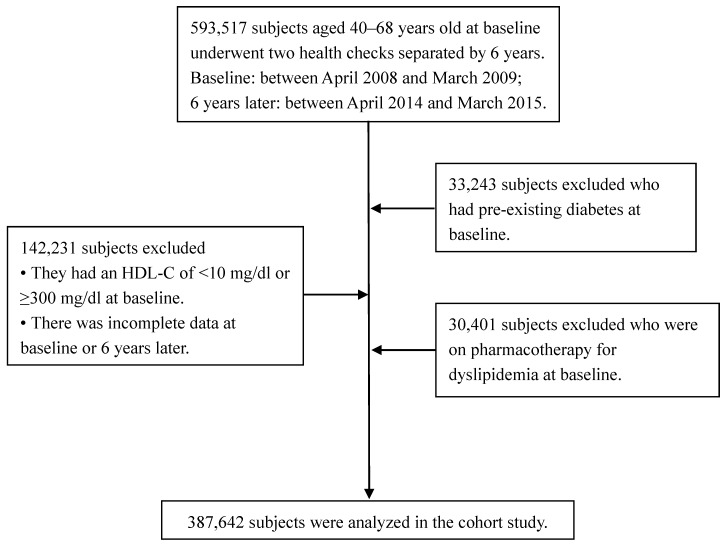
Exclusion criteria and subject disposition in the study.

This cohort study used data collected from people who attended health checks twice 6 years apart: between April 2008 and March 2009, and between April 2014 and March 2015. The inclusion and exclusion criteria are shown in Figure 1. A total of 387,642 people (219,047 men and 168,595 women) aged 40–68 years at baseline satisfied these criteria and were enrolled in the study. People aged 69–74 years at baseline were not enrolled because the data of such people after six years were unavailable. Subjects with pre-existing diabetes, those undergoing pharmacotherapy for dyslipidemia, and those residing in medical institutions, including hospitals and nursing homes, were not included. However, some of the subjects included had undergone pharmacotherapy for hypertension, and some had a history of CVD. All of these conditions were digitally recorded as answers in a questionnaire.

To evaluate subject age as a single numeric value, we transformed the age groups (40–44, 45–49, 50–54, 55–59, 60–64, and 65–69 years) into substituted ages (s-age), corresponding to the median for each age group (42, 47, 52, 57, 62, and 67 years, respectively). Subjects were also classified into nine categories according to their baseline HDL-C concentration: ≤39, 40–49, 50–59, 60–69, 70–79, 80–89, 90–99, 100–109, and ≥110 mg/dL, which were ~10 mg/dL higher than the categories used in a previous study [20], because the HDL-C of Japanese people tends to be ~10 mg/dL higher than that of people in western countries [27] and the values in Japan were likely to have increased over the previous two decades [28]. Following the annual health check, subjects with or at risk of metabolic syndrome are expected to undergo health guidance provided in-house or by outsourced healthcare agencies [25,26], although this is not obligatory. Such health guidance can be classified into two categories, intensive or motivational, depending on the outcomes of the health check [26]. Intensive health guidance involves follow-up consultations by e-mail, phone, or face-to-face for up to 6 months, as previously described [25].

### 2.3. Measurements

Body mass index (BMI) was calculated as mass (kg) divided by the square of height (m). Biochemical measurements were performed automatically using standard methods. Serum low-density lipoprotein cholesterol (LDL-C), HDL-C, and triglyceride (TG) concentrations were measured automatically, mainly (in ~85% of samples) spectrophotometrically (using a direct, non-precipitation method) [29,30], and the remainder were measured using other methods, following rigorous instructions from the MHLW [25]. Fasting plasma glucose (FPG) was mainly measured (~54% of samples) spectrophotometrically or potentiometrically (~30%). HbA1c was mainly measured using an immunoassay (65%) or high-performance liquid chromatography (17%) [25].

All subjects had either or both their HbA1c and FPG measured (65% and 72% of subjects had both measured at baseline and 6 years later, respectively), in addition to providing information about any pharmacotherapy for diabetes. Both pre-existing diabetes at baseline and diabetes after 6 years of the study were defined by the presence of HbA1c ≥ 6.5%, FPG ≥ 126 mg/dL, and/or the use of pharmacotherapy for diabetes. Most of the diabetes identified in this study was considered to be type 2 diabetes, because this is much more prevalent in middle-aged and older people [24], although the type was not confirmed.

### 2.4. Statistical Analysis

Data are expressed as the mean ± SD, or median (interquartile range). Continuous and categorical variables were compared between HDL-C groups using analysis of variance (ANOVA) or χ^2^-tests, respectively. The incidence of diabetes per 1000 person-years was determined according to baseline HDL-C category. A logistic regression model was used to evaluate the relationship between baseline HDL-C category and the incidence of diabetes, with adjustment for potential confounding factors, which yielded adjusted relative risks (ARR) and 95% confidence intervals (CIs), because the incidence of diabetes was small (3.8% in total), meaning that adjusted odds ratios are near equivalent to ARRs. These associations were adjusted according to initial age and sex. Sequentially, additional adjustments for confounders at baseline were conducted, which were current smoking, alcohol intake (amount and frequency), exercise habits, use of pharmacotherapy for hypertension, BMI, and FPG. Additionally, a traditional linear model with an identity-link function via a generalized linear model was used to evaluate associations between HbA1c and FPG as continuous variables, which are unaffected by the criteria of diabetes, and baseline HDL-C categories. Statistical analysis was performed using SAS-Enterprise Guide (SAS-EG 7.1) in SAS software, version 9.4 (SAS Institute, Cary, NC, USA). *p* < 0.05 was considered to represent statistical significance.

## 3. Results

Table 1 shows the subject characteristics at baseline, classified according to the nine baseline HDL-C categories. While the median HDL-C concentration was 63 mg/dL in total (57 mg/dL for men and 71 mg/dL for women), there were 12,908 subjects with HDL-C ≥100 mg/dL (3.3% of the total). s-Age and the prevalence of women, regular exercise, and daily alcohol intake were higher in the higher HDL-C groups than in the lower HDL-C groups, whereas BMI, blood pressure, TG, LDL-C, HbA1c, and FPG were lower (by ANOVA or *χ*^2^-test; all *p* < 0.0001). Pharmacotherapy for hypertension, past history of CVD, and current smoking were less prevalent in higher HDL-C groups (*χ*^2^-test; all *p* < 0.0001). However, the prevalence of heavy alcohol intake (≥69 g ethanol/day) showed a U-shaped relationship with HDL-C, with a minimum at 80–89 mg/dL HDL-C.

Table 2 shows the incidence of diabetes, the use of pharmacotherapy, and other measurements after 6 years, classified according to baseline HDL-C category. Diabetes was recorded in 14,802 subjects (3.8% of the total), and the lowest incidences were recorded in men with a baseline HDL-C of 80–89 mg/dL (4.8/1000 person-years) and women with a baseline HDL-C of 100–109 mg/dL (1.5/1000 person-years).

On the whole, the incidence of diabetes was higher in heavy alcohol drinkers than in mild-to-moderate alcohol drinkers in all HDL-C groups. The prevalence of pharmacotherapy for diabetes, hypertension, or dyslipidemia, and the concentrations of FPG and HbA1c, after 6 years, were lower in subjects with higher baseline HDL-C (all *p* < 0.0001; *χ*^2^-test or ANOVA), although there was a slightly higher prevalence of pharmacotherapy for hypertension in the highest HDL-C group (15.8%). In addition, the concentrations of HDL-C in the lower and higher baseline HDL-C categories were closer to the average values across all the subjects after 6 years.

Table 3 shows the results of logistic regression analysis. An HDL-C of 80–89 mg/dL was used as the provisional reference, having considered the lowest incidences of diabetes among the nine groups (Table 2). Compared with subjects who had an HDL-C of 80–89 mg/dL, those with an HDL-C <70 mg/dL were at significantly greater risk of diabetes (Model 2). However, after adjustment for confounding factors in addition to age and sex, subjects with an HDL-C ≥110 mg/dL were also shown to be at greater risk of diabetes, consistent with a concave relationship between ARR and diabetes incidence (Model 3), which was not substantially changed after participants were classified into mild-to-moderate or heavy alcohol drinkers. However, when the analysis was restricted to subjects who rarely drank alcohol (*n* = 145,410), those with an HDL-C of ≥110 mg/dL were not at greater risk of diabetes (1.42 (0.89–2.27)). When baseline FPG and HbA1c were further adjusted according to sex, men with an HDL-C of ≥100 mg/dL were shown to be at significantly greater risk of diabetes, whereas female subjects needed an HDL-C of ≥110 mg/dL to be at greater risk (Model 4). When subjects were restricted to those who were not on pharmacotherapy for dyslipidemia after six years (Model 3), the ARR of subjects with an HDL-C of ≥110 mg/dL was rather strengthened (1.60 (1.27–2.00), *p* < 0.0001).

Table 4 shows the results of the generalized linear model for HbA1c and FPG as a continuous variable. Before adjustment for BMI (Model 1 and Model 2), high HDL-C of ≥100 mg/dL was inversely associated with HbA1c, which was not statistically significant after adjustment for BMI (Model 3). After full adjustment for confounders (Model 5), HDL-C of 90–99 mg/dL and of ≥110 mg/dL were positively associated with HbA1c. Similarly, after adjustment for BMI (Model 3), high HDL-C of ≥90 mg/dL was positively associated with FPG. However, after full adjustment for confounders (Model 5), only HDL-C of ≥110 mg/dL was significantly associated with FPG (Model 5).

## 4. Discussion

Several studies conducted in recent years have shown that extremely high serum HDL-C may not be protective against the development of CVD [20,21,22], although the underlying mechanism remains to be established. The findings of the present study suggest that individuals with extremely high HDL-C, around 110 mg/dL, may be at greater risk of diabetes, like those with a low HDL-C, suggesting a potential effect of high HDL on glucose metabolism. Alternatively, the present results suggest a possibility of extremely high HDL-C as a potential marker for diabetes and that individuals with extremely high HDL-C may be at an unfavorable state in terms of a relatively higher incident of diabetes, although the underlying mechanism may be complicated, which includes BMI and alcohol consumption.

This finding was made for the first time using a very large database of health check data obtained for people across the entire Kanagawa prefecture, which has the second-largest population in Japan, next to Tokyo. Furthermore, we have determined a provisional reference level of HDL-C, which, at least in terms of diabetes risk, may be higher than previously used for CVD: possibly around 80–89 mg/dL, rather than 60–70 mg/dL [15,20]. Intriguingly, and consistent with our results, two previous studies conducted in Japan [22,31] have shown that individuals with a high HDL-C (≥90 or 100 mg/dL) were more likely to have diabetes or high FPG (≥100 mg/dL) than those with an HDL-C of 80–89 mg/dL, although statistically significant differences were not described.

Thus, a concentration of HDL-C of >80 mg/dL, which has been conventionally considered to be very high, may not actually be “very high”, but instead only relatively high or indeed an optimal concentration of HDL-C. In addition, previous clinical studies that have been considered large were unlikely to be sufficiently powered for the detailed analysis of participants with very high HDL-C (e.g., ≥80 mg/dL) [15,18], meaning that the categories in our study that represent a provisional optimal HDL-C and an extremely high HDL-C had been previously combined. Consequently, the impact of extremely high HDL-C on the incidence of CVD could not be appropriately evaluated.

In our study, the proportion of subjects with an HDL-C of ≥100 mg/dL at baseline (3.3%) was higher than in previous studies, yielding an adequate sample size of 12,908 for the group with extremely high HDL-C for reliable analysis. However, the proportion of subjects with an HDL-C of ≥100 mg/dL in our study was close to those of studies conducted in recent years by Ko et al. (2.8%) [20], Madsen et al. (possibly ~3.0%) [21], and Yoshida et al. (3.1%) [32].

For the past decade, many clinical studies have shown that the usage of statin, a first-line therapy for high LDL-C, can increase the risk of diabetes [33,34,35]. However, the prevalence of subjects with pharmacotherapy for dyslipidemia was lowest in the highest HDL-C group (5%) in this study (Table 2). In addition, when subjects were restricted to those without pharmacotherapy for dyslipidemia, the ARR of HDL-C of ≥110 mg/dL was not attenuated (Table 3). Therefore, high HDL-C and the relatively high incidence of diabetes in the highest HDL-C group are unlikely to be attributable to the usage of statin.

Numerous studies have demonstrated that people with low HDL-C are predisposed to type 2 diabetes [24], and a Japanese cohort study also showed a greater risk of diabetes in people with low HDL-C [36]. Consistent with these, our present data demonstrate that people with low HDL-C are at greater risk of developing diabetes within 6 years. HDL particles can enhance glucose uptake by skeletal muscle and stimulate insulin secretion from pancreatic β cells [37]. However, this may only occur if HDL function is normal, and its anti-atherosclerotic, anti-diabetic, anti-inflammatory, and immunologic [13,37] effects might be impaired in subjects with extremely high HDL-C.

In addition to the quantity and quality of HDL, high alcohol intake, slight hypertension, or smoking might combine to predispose towards diabetes, although these potential confounders were adjusted for in the statistical analyses conducted in this study. To date, the clinical profile of people with high HDL-C has been poorly characterized. In this study, the lipid profile and BMI of the highest HDL-C group seemed to be the best (with the lowest levels of TG, LDL-C, and BMI) of the nine HDL-C groups. In addition, subjects who habitually exercised to a light sweat were much more frequent in the highest HDL-C group. However, the profile was less favorable with regard to other known CVD risks: this group were older, and had a higher prevalence of heavy and/or daily alcohol intake, slightly higher prevalence of smoking, slight hypertension, and/or slightly higher prevalence of pharmacotherapy for hypertension. Taken together, the features of subjects with very high HDL-C may be heterogeneous, consisting of both sides of healthy and unhealthy elements.

Similar trends regarding other CVD risks were observed in individuals with extremely high HDL-C in studies by Ko et al. [20], Hirata et al. [22], and Moriyama et al. [31], although the authors did not refer to them. The blood pressure findings might be consistent with the results of clinical trials that evaluated the effect of cholesterol ester transfer protein (CETP) inhibitors (torcetrapib and evacetrapib), which increased plasma HDL-C by 133% over 12 months, but also increased systolic blood pressure, on CVD events [38,39]. In these studies, CETP inhibitors likely increased serum HDL-C beyond 100 mg/dL in a large proportion of patients in the test arm. However, such an increase in HDL-C would have been unlikely to mediate the protection against CVD events, although the underlying mechanism and the incidence of diabetes in this sample are unknown.

In our study, the prevalences of daily and heavy alcohol intake were both highest in patients with HDL-C ≥110 mg/dL (44.9% and 5.6%, respectively) among the nine HDL-C groups (Table 1), although we had no information regarding the type of alcoholic beverage consumed. In general, a J-shaped relationship between alcohol intake and plasma TG has been shown [40,41]. Intriguingly, however, the serum TG in participants with an HDL-C ≥110 mg/dL was the lowest, despite their high and frequent alcohol consumption. Seike et al. [42] reported in a systematic review that alcohol intake may be a risk factor for type 2 diabetes, especially in Japanese people with a low BMI (≤22.0 kg/m^2^), although the mechanism remains to be established. In our study, subjects with HDL-C >110 mg/dL had the lowest BMI and consumed alcohol more heavily and frequently. In addition, after adjustment for BMI, the associations of HbA1c and FPG with high HDL-C were changed (Table 3 and Table 4). Therefore, a common mechanism may be at work.

Another major cause of high serum HDL-C may be CETP deficiency arising due to *CETP* gene mutations [6,7,8]. Unfortunately, the causes of the extremely high HDL-C in subjects in this study are unclear because gene sequencing and measurement of CETP activity were not conducted. However, it has been reported that in half of individuals with high HDL-C in Japan a *CETP* gene mutation is responsible [43].

Several limitations of the study should be mentioned. First, the type of diabetes, for instance, type 2, type 1, or secondary, was not confirmed in this study, although type 2 diabetes is the most prevalent in the general population [24]. Second, HDL-C was measured by direct methods, rather than precipitation methods, which may affect the precision of the measurement and prevent comparison with previous data obtained using precipitation methods. However, it has been reported that most direct methods of HDL-C assay (six of eight) met the National Cholesterol Education Program total error goals for healthy individuals [30]. Third, the function of HDL, the subclass Apo A-I expressed, and the secondary causes of high HDL-C, including liver disease and hormone therapy in women, were not evaluated in this study. Fourth, the genetic background of the participants with respect to gene mutations, such as *CETP* and *HTGL*, was also unknown. Finally, as described above, the Japanese population has a higher concentration of HDL-C than the U.S. population [27], and this has been increasing over the past two decades [28]. Therefore, the current conclusions may not be applicable to populations who have lower serum HDL-C concentrations.

In conclusion, individuals with extremely high HDL-C may be at greater risk of diabetes, like those with low HDL-C, and the optimal level for serum HDL-C, at least with regard to the prevention of diabetes, may be higher than previously thought.

## Figures and Tables

**Table 1:** Characteristics of subjects at baseline.

HDL-C Category (mg/dL)	≤39	40–49	50–59	60–69	70–79	80–89	90–99	100–109	≥110
N (% in total)	13,718 (3.54)	58,284 (15.0)	90,133 (23.3)	87,922 (22.7)	66,136 (17.1)	39,121 (10.1)	19,420 (5.01)	7983 (2.06)	4925 (1.27)
s-Age (years old)	51.2 ± 8.4	51.5 ± 8.4	52.1 ± 8.6	52.4 ± 8.7	52.6 ± 8.6	52.9 ± 8.5	53.0 ± 8.3	53.4 ± 8.2	53.6 ± 8.1
Women, *n* (%)	1351 (9.9)	10,082 (17.3)	27,595 (30.6)	40,884 (46.5)	39,303 (59.4)	26,420 (67.5)	13,779 (71.0)	5732 (71.8)	3449 (70.0)
BMI (kg/m^2^)	25.2 ± 3.2	24.5 ± 3.1	23.5 ± 3.0	22.5 ± 2.9	21.7 ± 2.7	21.0 ± 2.6	20.7 ± 2.5	20.4 ± 2.4	20.3 ± 2.4
SBP (mmHg)	124 ± 16	124 ± 16	123 ± 16	122 ± 17	121 ± 17	120 ± 17	120 ± 17	121 ± 17	122 ± 17
DBP (mmHg)	78 ± 11	78 ± 11	77 ± 11	76 ± 11	75 ± 11	74 ± 11	74 ± 11	74 ± 11	75 ± 11
HDL-C (mg/dL)	36 ± 3.0	45 ± 2.8	55 ± 2.8	64 ± 2.9	74 ± 2.8	84 ± 2.8	94 ± 2.8	104 ± 2.8	121 ± 12.5
LDL-C (mg/dL) *	125 ± 31	133 ± 30	133 ± 31	128 ± 31	123 ± 30	120 ± 30	116 ± 29	114 ± 30	108 ± 31
TG (IQ) (mg/dL)	185 (133–260)	141 (102–198)	108 (79–150)	87 (65–119)	74 (56–100)	67 (52–89)	63 (49–82)	60 (48–79)	59 (47–76)
HbA1c (%) (available *n* = 315,506)	5.5 ± 0.4	5.5 ± 0.3	5.4 ± 0.3	5.4 ± 0.3	5.4 ± 0.3	5.4 ± 0.3	5.4 ± 0.3	5.4 ± 0.3	5.4 ± 0.3
FPG (mg/dL) (available *n* = 321,001)	96 ± 9.6	95 ± 9.5	94 ± 9.3	93 ± 9.2	92 ± 9.0	91 ± 8.9	91 ± 9.0	91 ± 9.1	92 ± 9.3
Pharmacotherapy for hypertension, *n* (%)	1874 (13.7)	7634 (13.1)	10,591 (11.8)	9016 (10.3)	5774 (8.7)	3021 (7.7)	1395 (7.2)	547 (6.9)	371 (7.5)
CVD, *n* (%)	350 (2.6)	1333 (2.3)	2084 (2.3)	1890 (2.2)	1240 (1.9)	730 (1.9)	377 (1.9)	128 (1.6)	81 (1.6)
Smokers, *n* (%) ^†^	6786 (49.5)	22,494 (38.6)	26,676 (29.6)	19,627 (22.3)	11,707 (17.7)	5901 (15.1)	2777 (14.3)	1089 (13.6)	764 (15.5)
Exercisers, *n* (%) ^‡^	2793 (20.4)	13,609 (23.4)	23,323 (25.9)	24,428 (27.8)	19,525 (29.5)	12,213 (31.2)	6294 (32.4)	2812 (35.2)	1810 (36.8)
Alcohol intake									
Frequency									
Almost none, *n* (%)	6049 (44.1)	22,306 (38.3)	33,117 (36.7)	33,039 (37.6)	25,172 (38.1)	14,848 (38.0)	6883 (35.4)	2643 (33.1)	1353 (27.5)
Daily, *n* (%)	2660 (19.4)	15,436 (26.5)	27,307 (30.3)	27,240 (31.0)	20,335 (30.8)	12,343 (31.6)	6615 (34.1)	3033 (38.0)	2210 (44.9)
Amount									
Light, *n* (%) ^§^	8764 (63.9)	35,207 (60.4)	53,579 (59.4)	54,373 (61.8)	42,622 (64.5)	25,624 (65.5)	12,356 (63.6)	4809 (60.2)	2695 (54.7)
Heavy, *n* (%) ^¶^	631 (4.6)	2724 (4.7)	3895 (4.3)	3559 (4.1)	2433 (3.7)	1280 (3.3)	662 (3.4)	327 (4.1)	274 (5.6)

* Available *n* = 386,590. ^†^ Current regular smoker. ^‡^ Exercise to a light sweat for over 30 min per session, twice weekly. ^§^ Alcohol intake corresponding to <23 g ethanol per day. ^¶^ Alcohol intake corresponding to >69 g ethanol per day. Significant differences in systolic blood pressure and prevalence of pharmacotherapy for hypertension were observed between the 80–89 mg/dL and ≥110 mg/dL HDL-C groups (post hoc analysis: Bonferroni; *p* < 0.001). Significant differences in the proportions of heavy alcohol drinkers were observed between the 80–89 mg/d and the other HDL-C groups, except for the 90–99 mg/dL group (*χ*^2^-test; all *p* < 0.001). BMI, body mass index; SBP, systolic blood pressure; DBP, diastolic blood pressure; HDL-C, high-density lipoprotein cholesterol; LDL-C, low-density lipoprotein cholesterol; TG, triglyceride; FPG, fasting plasma glucose; CVD, cardiovascular disease.

**Table 2:** Incidence of diabetes and use of pharmacotherapy, and other measured variables, after 6 years, classified according to baseline HDL-C category.

Baseline HDL-C Category (mg/dL)	≤39	40–49	50–59	60–69	70–79	80–89	90–99	100–109	≥110
Incidence of diabetes per 1000 person-years									
Total	14.7	11.2	7.7	5.3	3.8	2.8	2.7	2.5	3.5
Men	15.1	11.5	8.5	6.6	5.5	4.8	5.0	4.9	7.1
Women	11.2	9.7	6.1	3.8	2.6	1.9	1.8	1.5	1.9
Alcohol consumption									
Mild-to-moderate alcohol drinkers *	14.4	10.9	7.4	4.9	3.4	2.5	2.3	2.2	2.7
Heavy alcohol drinkers ^†^	16.7	12.8	9.4	7.4	6.1	4.7	5.5	3.6	6.1
Pharmacotherapy									
Diabetes, *n* (%)	414 (3.0)	1283 (2.2)	1313 (1.5)	851 (1.0)	449 (0.7)	207 (0.5)	96 (0.5)	44 (0.6)	30 (0.6)
Hypertension, *n* (%)	3443 (25.1)	14,255 (24.5)	19,862 (22.0)	16,901 (19.2)	10,941 (16.5)	5883 (15.0)	2856 (14.7)	1160 (14.5)	777 (15.8)
Dyslipidemia, *n* (%)	1863 (13.6)	7488 (12.9)	10,355 (11.5)	8653 (9.8)	5296 (8.0)	2666 (6.8)	1153 (5.9)	440 (5.5)	245 (5.0)
HDL-C (mg/dL)	41.1 ± 7.8	48.5 ± 8.3	56.5 ± 9.2	65.0 ± 10.2	73.2 ± 11.1	81.2 ± 12.1	88.7 ± 13.6	96.2 ± 15.1	106 ± 21.4
FPG (mg/dL)	101 ± 18	99 ± 15	97 ± 14	95 ± 13	94 ± 12	93 ± 11	93 ± 11	93 ± 11	94 ± 11
HbA1c (%)	5.7 ± 0.6	5.6 ± 0.5	5.6 ± 0.5	5.5 ± 0.4	5.5 ± 0.4	5.5 ± 0.3	5.5 ± 0.3	5.5 ± 0.3	5.5 ± 0.4

* equivalent to consumption of <46 g ethanol per session (*n* = 200,279). ^†^ equivalent to consumption of ≥46 g ethanol per session (*n* = 49,028).

**Table 3:** Relative risk (95% confidence interval) of incident diabetes after 6 years, classified according to baseline HDL-C category.

Baseline HDL-C Category (mg/dL)	≤39	40–49	50–59	60–69	70–79	80–89	90–99	100–109	≥110
Model 1	5.65 (5.12–6.22) ^‡^	4.21 (3.87–4.57) ^‡^	2.84 (2.62–3.09) ^‡^	1.92 (1.76–2.09) ^‡^	1.35 (1.23–1.48) ^‡^	1 (ref)	0.97 (0.85–1.11)	0.88 (0.72–1.07)	1.23 (1.00–1.52)
Model 2	4.32 (3.91–4.77) ^‡^	3.32 (3.04–3.61) ^‡^	2.36 (2.17–2.56) ^‡^	1.72 (1.58–1.88) ^‡^	1.29 (1.18–1.42) ^‡^	1 (ref)	0.99 (0.87–1.13)	0.89 (0.73–1.08)	1.23 (1.00–1.52)
Model 3	1.56 (1.40–1.74) ^‡^	1.53 (1.39–1.67) ^‡^	1.38 (1.26–1.50) ^‡^	1.26 (1.15–1.37) ^‡^	1.12 (1.02–1.23) *	1 (ref)	1.08 (0.94–1.24)	1.04 (0.85–1.26)	1.46 (1.18–1.81) ^†^
Model 3 No pharmacotherapy for dyslipidemia (available *n* = 349,313)	1.60 (1.41–1.81) ^‡^	1.50 (1.35–1.66) ^‡^	1.38 (1.25–1.52) ^‡^	1.26 (1.14–1.39) ^‡^	1.13 (1.02–1.26) *	1 (ref)	1.10 (0.94–1.27)	1.03 (0.83–1.29)	1.60 (1.27–2.00) ^‡^
Model 3 Mild-to-moderate alcohol drinkers ^§^ (available *n* = 328,826)	1.53 (1.35–1.72) ^‡^	1.54 (1.39–1.71) ^‡^	1.41 (1.28–1.56) ^‡^	1.27 (1.15–1.41) ^‡^	1.12 (1.01–1.25) *	1 (ref)	1.01 (0.86–1.19)	1.08 (0.86–1.35)	1.35 (1.03–1.75) *
Model 3 Heavy alcohol drinkers ^||^ (available *n* = 58,816)	1.67 (1.31–2.12) ^‡^	1.45 (1.19–1.76) ^†^	1.26 (1.04–1.51) *	1.20 (1.00–1.45)	1.13 (0.92–1.38)	1 (ref)	1.29 (0.98–1.69)	0.90 (0.60–1.36)	1.63 (1.13–2.36) ^†^
Model 4 (available *n* = 250,373)	1.49 (1.28–1.73) ^‡^	1.34 (1.18–1.51) ^‡^	1.26 (1.12–1.41) ^†^	1.17 (1.04–1.32) ^†^	1.16 (1.02–1.31) *	1 (ref)	1.11 (0.92–1.34)	1.25 (0.97–1.61)	1.93 (1.48–2.52) ^‡^
Model 4 Men (available *n* = 140,633)	1.43 (1.19–1.72) ^†^	1.26 (1.07–1.48) ^†^	1.18 (1.00–1.38) *	1.14 (0.97–1.33)	1.21 (1.02–1.43) *	1 (ref)	1.24 (0.95–1.60)	1.57 (1.10–2.24) *	2.45 (1.70–3.53) ^‡^
Model 4 Women (available *n* = 109,740)	1.49 (1.04–2.15) *	1.45 (1.18–1.80) ^†^	1.34 (1.11–1.61) ^†^	1.15 (0.96–1.37)	1.06 (0.88–1.28)	1 (ref)	1.02 (0.78–1.32)	1.02 (0.70–1.48)	1.53 (1.02–2.29) *

* *p* < 0.05, ^†^ *p* < 0.01, ^‡^ *p* < 0.0001. Model 1: Unadjusted. Model 2: Adjusted for age and sex. Model 3: Model 2 plus adjustment for use of anti-hypertensive therapy, history of cardiovascular disease, frequency and quantity of alcohol consumption, and regular exercise (≥30 min exercise per session >2 times/week versus less frequent exercise), BMI, systolic blood pressure, triglyceride, and smoking status. Model 4: Model 3 plus adjustment for FPG and HbA1c. All the adjustments for potential confounding factors were made using the baseline values. ^§^ <46 g ethanol per day, ^||^ ≥46 g ethanol per day.

**Table 4:** Beta coefficients and 95% CIs for HbA1c and FPG according to baseline HDL-C category.

Baseline HDL-C Category (mg/dL)	≤39	40–49	50–59	60–69	70–79	80–89	90–99	100–109	≥110
HbA1c									
Model 1	0.21 (0.20–0.22) ***	0.16 (0.15–0.16) ***	0.10 (0.09–0.11) ***	0.05 (0.04–0.06) ***	0.02 (0.01–0.02) ***	0.0 (ref)	−0.01 (−0.02–0.00)	−0.02 (−0.03–−0.01) **	−0.01 (−0.03–0.00) *
Model 2	0.23 (0.22–0.24) ***	0.17 (0.17–0.18) ***	0.11 (0.10–0.12) ***	0.06 (0.05–0.06) ***	0.02 (0.01–0.03) ***	0.0 (ref)	−0.01 (−0.02–0.00)	−0.02 (−0.03–−0.01) **	−0.02 (−0.03–−0.01) **
Model 3	0.14 (0.13–0.15) ***	0.10 (0.09–0.10) ***	0.05 (0.05–0.06) ***	0.02 (0.02–0.03) ***	0.01 (0.00–0.01) *	0.0 (ref)	0.00 (−0.01–0.01)	0.00 (−0.01–0.01)	0.00 (−0.01–0.02)
Model 4	0.11 (0.10–0.11) ***	0.08 (0.07–0.08) ***	0.04 (0.04–0.05) ***	0.02 (0.01–0.02) ***	0.00 (0.00–0.01)	0.0 (ref)	0.01 (0.00–0.01)	0.00 (−0.01–0.02)	0.02 (0.00–0.03) *
Model 5	0.04 (0.03–0.05) ***	0.03 (0.03–0.04) ***	0.02 (0.01–0.02) ***	0.01 (0.00–0.01)	0.00 (−0.01–0.00)	0.0 (ref)	0.01 (0.00–0.02) *	0.01 (0.00–0.02)	0.02 (0.01–0.03) **
FPG									
Model 1	7.29 (7.00–7.58) ***	5.73 (5.54–5.92) ***	3.97 (3.79–4.14) ***	2.14 (1.96–2.32) ***	0.94 (0.75–1.12) ***	0.0 (ref)	−0.16 (−0.41–0.10)	−0.12 (−0.47–0.24)	0.36 (−0.08–0.81)
Model 2	4.53 (4.24–4.82) ***	3.32 (3.13–3.51) ***	2.14 (1.97–2.32) ***	1.10 (0.93–1.27) ***	0.54 (0.36–0.73) ***	0.0 (ref)	0.00 (−0.25–0.25)	0.00 (−0.35–0.35)	0.37 (−0.06–0.81)
Model 3	1.70 (1.40–1.99) ***	0.98 (0.79–1.18) ***	0.46 (0.29–0.64) ***	0.12 (−0.06–0.29)	0.12 (−0.06–0.29)	0.0 (ref)	0.27 (0.02–0.52) *	0.49 (0.15–0.84) **	1.00 (0.58–1.43) ***
Model 4	2.51 (2.21–2.80) ***	1.57 (1.37–1.77) ***	0.84 (0.66–1.02) ***	0.32 (0.15–0.50)	0.20 (0.02–0.38) *	0.0 (ref)	0.17 (−0.08–0.41)	0.27 (−0.08–0.61)	0.61 (0.19–1.04) **
Model 5	1.03 (0.72–1.34) ***	0.64 (0.43–0.85) ***	0.35 (0.17–0.53) ***	0.10 (−0.07–0.27)	0.12 (−0.06–0.30)	0.0 (ref)	0.19 (−0.06–0.43)	0.30 (−0.05–0.64)	0.61 (0.18–1.03) **

* *p* < 0.05, ** *p* < 0.01, *** *p* < 0.0001. Model 1: Unadjusted. Model 2: Adjusted for age and sex. Model 3: Model 2 plus adjustment for BMI. Model 4: Model 3 plus adjustment for frequency and quantity of alcohol consumption. Model 5: Model 4 plus adjustment for use of anti-hypertensive therapy, history of cardiovascular disease, and regular exercise (≥30 min exercise per session >2 times/week versus less frequent exercise), systolic blood pressure, triglyceride, and smoking status. All the adjustments for potential confounding factors were made using the baseline values. ^§^ <46 g ethanol per day, || ≥46 g ethanol per day.
